# The relationship between processing speed and remodeling spatial patterns of intrinsic brain activity in the elderly with different sleep duration

**DOI:** 10.3389/fnins.2023.1185078

**Published:** 2023-05-26

**Authors:** Li Pu, Yao Zou, Yan Wang, Jia-Ling Lei, Xiao-Nan Zhao, Xia Zeng, Guo-Jian Yan

**Affiliations:** ^1^The Fourth People’s Hospital of Chengdu, Chengdu, China; ^2^Department of Neurology, Chengdu 363 Hospital, Chengdu, China

**Keywords:** short sleep duration, amplitude of low frequency fluctuations, regional homogeneity, degree centrality, processing speed

## Abstract

**Objective:**

Brain neuroplasticity in which sleep affects the speed of information processing in the elderly population has not been reported. Therefore, this study was conducted to explore the effects of sleep on information processing speed and its central plasticity mechanism in the elderly.

**Methods:**

A total of 50 individuals aged 60 and older were enrolled in this case control study. All subjects were divided into two groups according to the sleep time: short sleep duration (< 360 min) (6 men and 19 women; mean age: 66.96 ± 4.28 years old), and non-short sleep duration (> 360 min) (13 men and 12 women). Resting-state functional magnetic resonance imaging (rs-fMRI) data were collected, and the amplitude of low frequency fluctuation (ALFF), regional homogeneity (ReHo), and degree centrality (DC) were calculated for each participant. Two-sample *t*-tests were performed to compare the ALFF, ReHo, and DC maps between the two groups. Then, the relationships among clinical features, fMRI and cognitive function were analyzed using general linear model.

**Results:**

Short sleep duration group showed significantly increased ALFF value in the bilateral middle frontal gyrus and right insula; significantly increased ReHo value in the left superior parietal gyrus, and decreased ReHo value in the right crebellum; significantly decreased DC value in the left inferior occipital gyrus, left superior parietal gyrus and right cerebellum (*p* < 0.05, AlphaSim correction). The ALFF value of right insula is significantly associated with symbol digit modalities test (SDMT) score (*β* = −0.363, *p* = 0.033).

**Conclusion:**

Short sleep duration and processing speed are significantly associated with remodeling spatial patterns of intrinsic brain activity in the elderly.

## 1. Introduction

Sleep problems are very common in the elderly, about 40% of people aged 60 or over suffer from sleep disorders, and this number increases with age (Luo et al., 2013). There is a clear relationship between the sleep habits of the elderly and chronic diseases, especially cognitive disorders (Winer et al., 2021). Sleep helps maintain the homeostasis of metabolism through immunity, immune support and hormones (Aldabal and Bahammam, 2011). Broken homeostasis causes the loss of neurons and the accumulation of toxic proteins, such as greater Aβ burden in the brain, which are some of the important reasons of the cognitive decline especially memory (Pan and Rickard, 2015; Lewis, 2021). Sleep is divided into two stages, non-rapid-eye-movement (NREM) and rapid-eye-movement (REM). As sleep enters NREM, a number of different electroencephalogram (EEG) patterns emerge, including spindle and slow waves, with clear links to memory. During sleep, the clearance of molecules such as Aβ is much higher in the brain than during wakefulness (Xie et al., 2013). Waste production rates also differ between waking states, with higher tau and Aβ production during wakefulness in rodents and humans (Holth et al., 2019). Thus, sleep can act as a pause in the waste production process, allowing time for the clearance system to catch up with the debris accumulated during wakefulness (Lewis, 2021).

Functional magnetic resonance imaging (fMRI) is a noninvasive technique that has been widely used to investigate central plasticity or neural plasticity in patients with neurological or psychiatric disorders. This technique provides valuable evidence of the brain’s ability to adapt and reorganize in response to various stimuli or injuries. Since its introduction, fMRI has revolutionized the field of neuroimaging and has become an indispensable tool for researchers and clinicians. By measuring changes in blood flow and oxygenation levels in the brain, fMRI can reveal important insights into the underlying neural mechanisms of various cognitive, emotional, and motor functions. Furthermore, fMRI has the potential to aid in the diagnosis and treatment of neurological and psychiatric disorders by identifying aberrant patterns of neural activity and predicting treatment outcomes (Lin et al., 2023; Wang et al., 2023). Therefore, fMRI represents a promising avenue for the future of neuroscience research and clinical practice (Logothetis, 2008). In addition, the influence of sleep on brain function is gradually known with the development of fMRI technology. REM sleep and slow wave sleep mediate critical cognitive and motor network functional activity to support enhanced offline performance associated with memory activation (Cousins et al., 2016). Memory undergoes a transitional process after encoding, which allows the hippocampus to rapidly acquire new information and store it stably in the neocortical long-term network, thus protecting the memory from interference. Sleep helps the process of long-term memory consolidation (Himmer et al., 2019). Another fMRI study reported that replay in the human hippocampus prioritizes weak knowledge, predicts subsequent memory performance, and is associated with improved memory in sleep latency (Schapiro et al., 2018). Sleep plays a crucial role in consolidating newly acquired memories. Brain imaging data show that functional interactions between dopaminergic reward areas, prefrontal cortex and hippocampus contribute to the integration of reward-associated memories. In particular, the sleep spindle enhances memory representations based on reward values, suggesting that privileged replay of information produces positive results (Igloi et al., 2015). Insomnia is the most common sleep disorder that causes disruption of the brain’s functional connectivity groups, which in turn leads to memory loss (Fasiello et al., 2022).

Brain neuroplasticity in which sleep affects memory function has been well studied, however, brain neuroplasticity in which sleep affects the speed of information processing in the elderly population has not been reported. Disruptions in sleep physiology can lead to age-related cognitive decline (Mander et al., 2014). Poor sleep can affect executive function and processing speed (Targa et al., 2021). Processing speed is an important mediator of the relationship between age and cognitive performance, and it influences the speed and outcome of many cognitive operations (Garrett et al., 2013). Therefore, our hypothesis is that short sleep may affect the brain function of elderly people, and this change in brain function may be related to cognitive function. This study was conducted to explore the effects of sleep on information processing speed and its central plasticity mechanism in the elderly. Our study not only fills in the brain plasticity mechanism of information processing speed changes in short-sleeping elderly population, but also provides potential neuromodulatory stimulation targets for this population.

## 2. Materials and methods

### 2.1. Subjects

A total of 50 individuals aged 60 and older were enrolled in this case control study. The population was homogeneous regarding race (Han nationality) and diet (dietary structure based on plant raw materials). The study was approved by the local ethics committee at the fourth people’s hospital of Chengdu from April 2021 to March 2022, and all patients were provided with written informed consent according to the Helsinki Declaration. All subjects underwent a neuropsychological assessment and a resting state fMRI (rs-fMRI) scan.

The inclusion criteria for all individuals was as follows: (1) Participants aged ≥60 years, (2) Mini-Mental State Examination (MMSE) score (Folstein et al., 1975) ≥ 24/30, (3) modified Rankin Scale (mRS) score (Bruno et al., 2010) ≤ 1, (4) no previous treatment for improving sleep and cognitive function, and (5) no cerebral parenchymal lesions.

The exclusion criteria for all individuals was as follows: (1) neurological diseases (e.g., any type of dementia, stroke, Parkinson’s disease, epilepsy), (2) systemic disease (e.g., cancer, severe abnormal glucose metabolism, serious heart, liver, kidney, blood system diseases, or infectious diseases), (3) psychiatric disease (e.g., anxiety, depression, schizophrenia), (4) severe hearing or visual impairment, (5) unsuitable for MRI, and (6) < 6 years of education.

All subjects were divided into two groups according to the sleep time: short sleep duration (< 360 min) (6 men and 19 women; mean age: 66.96 ± 4.28 years old), and non-short sleep duration (> 360 min) (13 men and 12 women; mean age: 66.04 ± 3.87 years old) (Da Silva et al., 2016). Sleep duration was determined according to the following two questions (Zhong et al., 2022): (1) How many minutes of actual sleep on average did you get at night (this may be shorter than the number of hours you spent in bed) during the past 6 months? and (2) How many minutes on average did you take a nap after lunch during the past 6 months?

### 2.2. Neuropsychological evaluation

Two neuropsychologists with >10 years of work experience performed the neuropsychological evaluation; another senior neuropsychologist then reviewed the assessment results. Processing speed was assessed by the Symbol Digit Modalities Test (SDMT) (Wang et al., 2012), written form was used in our study, subjects were given 1.5 min to match specific numbers with corresponding geometric figures. The number of correct responses within 1.5 min was recorded as the SDMT score, and higher scores indicate better performance. Visual search and sequencing were evaluated by the Shape Trail Test (STT) (Zhao et al., 2013). In the STT part B (STT_B), all numbers (from 1 to 25) were displayed and the participants are asked to make lines alternating between circles and squares and disregarding the numbers of the alternate shapes.

### 2.3. MRI data acquisition

MRI data were acquired using a MAGNETOM Verio 3.0-T scanner (Siemens Healthineers, Erlangen, Germany) with a 32-channel phased array head coil.

Light and sound shielding were applied before and during the scanning. Subjects were required to close their eyes and keep calm throughout the examination. Their heads were immobilized with foam pads, and their ears were plugged with earplugs. The rs-fMRI data were obtained using a single-pass gradient recalled echoplanar imaging (EPI) sequence with the following parameters: interleaved scanning order, repetition time (TR) = 3,000 ms, slice number = 43, matrix size = 64 × 64, transverse orientation, slice thickness = 3.0 mm, flip angle = 90°, gap = 0 (voxel size = 3.6 × 3.6 × 3.0 mm^3^), field of view (FOV) = 230 × 230 mm^2^, and number of acquisitions = 200.

### 2.4. Imaging data processing

Functional images of each subject were processed by the Resting-State fMRI Data Analysis Toolkit plus V1.2 (RESTplus V1.2, http://restfmri.net/forum/RESTplusV1.2), which is based on Statistical Parametric Mapping (SPM12)^1^ on the MATLAB 2013b (MathWorks, Natick, MA, USA) platform. The Data processing process is the same as the previous research (Ma et al., 2021): (1) Slice scan time correction, (2) Head movement correction (the head movements were all less than 2.0 mm or 2.0 degrees in any direction), (3) Spatial normalization of the functional images via the standard EPI template, (4) Regression of nuisance variables, including the white matter and cerebral spinal fluid blood oxygen level dependent (BOLD) signal and the effects of head motion using six head motion profiles, (5) Spatial smoothing was performed before the amplitude of low frequency fluctuations (ALFF) (Zang et al., 2007) calculation and after regional homogeneity (ReHo) (Lv et al., 2019) and degree centrality (DC) (Zuo et al., 2012) calculation using a Gaussian kernel of 6 mm full width at half maximum, (6) Removal of linear trends, and (7) ALFF, ReHo and DC were calculated for the traditional low-frequency band (0.01–0.08 Hz).

### 2.5. Statistical analysis

SPSS (version 21.0; IBM, Armonk, NY, United States) was used to conduct our analyses in clinical data. Data are expressed as mean (standard deviation) for continuous variables and percentage for categorical variables. Two-sample *t* -test was used for continuous variables; and Chi-square test was used for categorical variables. Only clinical data findings with two-tailed *p* < 0.05 were considered significant.

The ALFF, ReHo and DC maps were compared between two groups. Two-sample t- test was used for the two groups (two-tailed). AlphaSim correction was used for multiple comparisons, which was performed using the RESTplus toolkit. The resulting statistical map was set at *p* < 0.01 (AlphaSim correction) with a combined individual voxel *p* < 0.01.

The relationships among clinical features, fMRI and cognitive function were analyzed using general linear model. A general linear model was fitted to assess the independent association between the age/education/body mass index (BMI)/sleep duration and the ALFF/ReHo/DC value (as the dependent variable). Another general linear model was fitted to assess the independent association between the ALFF/ReHo/DC value and SDMT/STT_B score (as the dependent variable).

## 3. Results

### 3.1. Clinical and demographic characteristics

There was no significant difference in gender, age, education, smoking, drinking, body mass index (BMI), MMSE, STT_B and SDMT between the two groups. The sleep duration was 280.80 ± 37.63 in short sleep duration group, and 465.60 ± 58.17 in non-short sleep duration group (*p* < 0.05) (Table 1).

**Table 1:** Demographic characteristics of the elderly with short sleep duration and non-short sleep duration.

Characteristics	Short sleep duration group *n* = 25	Non-short sleep duration group *n* = 25	*p* value
*Basic characteristics*			
Gender (*%* female)	76%	48%	0.08^b^
Age (y)	66.96 (4.28)	66.04(3.87)	0.43^a^
Education (y)	10.52(2.26)	10.08(1.91)	0.46^a^
Smoking (*%*)	12%	24%	0.46^b^
Drinking (*%*)	4%	8%	1.00^b^
BMI(kg/m^2^)	23.28 (2.46)	22.97 (2.11)	0.63^a^
Sleep duration	280.80 (37.63)	465.60 (58.17)	**0.00** ^a^
MMSE	26.44 (1.73)	26.96 (1.88)	0.32^a^
STT_B (s)	153.49 (66.42)	194.32 (88.32)	0.07^a^
SDMT	27.12 (9.35)	29.52 (13.35)	0.47^a^

a two sample *t* test.

b Chi-square test.

MMSE, Mini-Mental State Examination; STT_B, Shape Trail Test, part B; SDMT, Symbol Digit Modalities Test. Bold value: *p*<0.001.

### 3.2. ALFF analysis

**Figure 1:**
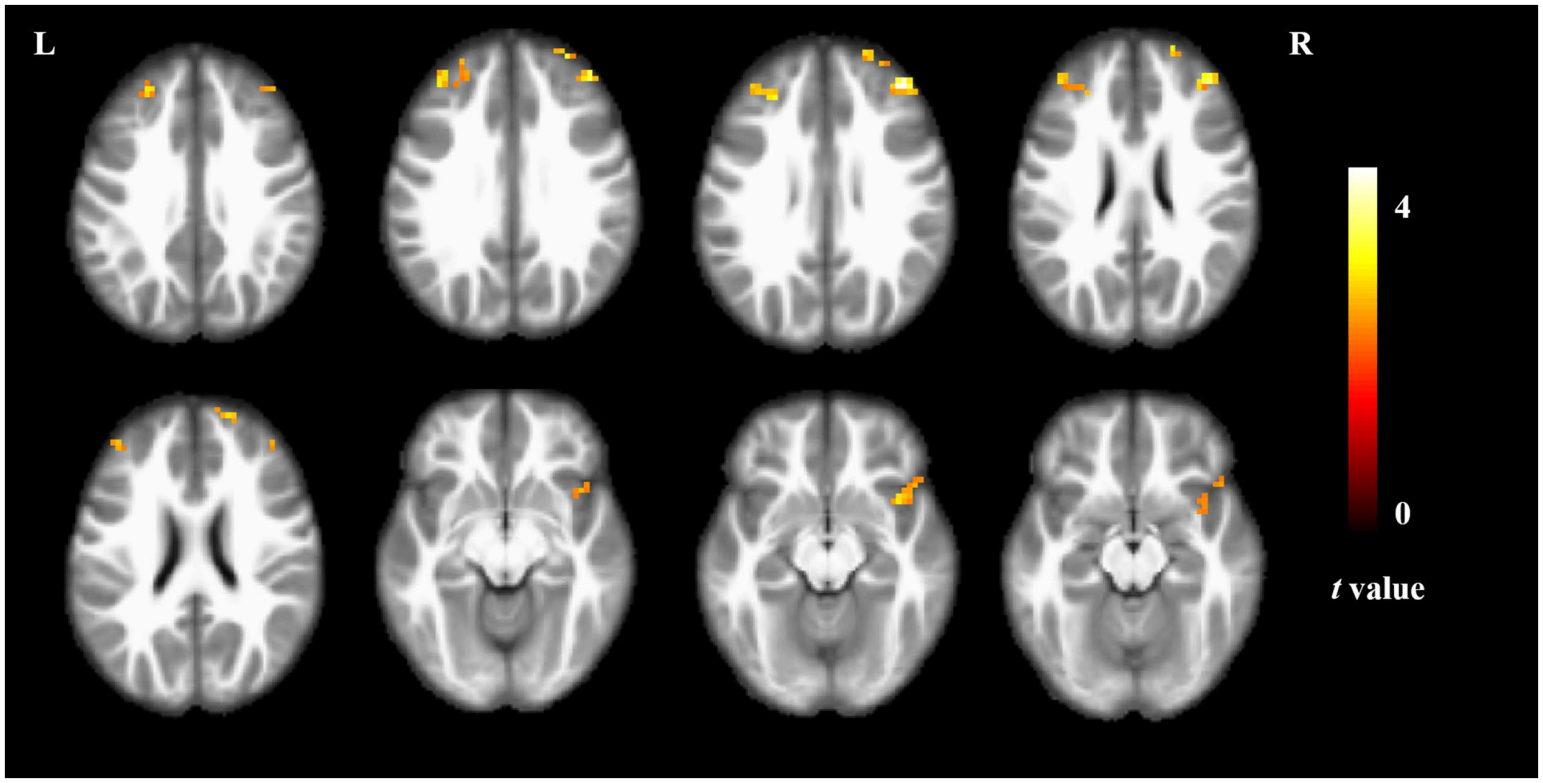
ALFF comparison between the elderly with short sleep duration and non-short sleep duration. The figure shows the two-sample *t*-test parameter diagram corrected by Alphasim. The warm color in color bar indicates that the ALFF value of the elderly with short sleep duration is higher than that of the non-short sleep duration group. The ALFF values of bilateral middle frontal gyrus and right insula increased in the elderly with short sleep duration.

Compared to non-short sleep duration group, short sleep duration group showed significantly increased ALFF value in the bilateral middle frontal gyrus and right insula (*p* < 0.05, AlphaSim correction, cluster size >21 voxels) (Figure 1 and Table 2).

**Table 2:** Brain region information of ALFF value.

Brain regions	Cluster size	Cluster centroid MNI coordinates			*t*-value
		x	y	z	
Right middle frontal gyrus	32	42	42	30	4.420
Left middle frontal gyrus	42	−27	36	30	3.355
Right insula	27	39	12	−12	3.107

MNI: Montreal Neurological Institute.

### 3.3. ReHo analysis

**Figure 2:**
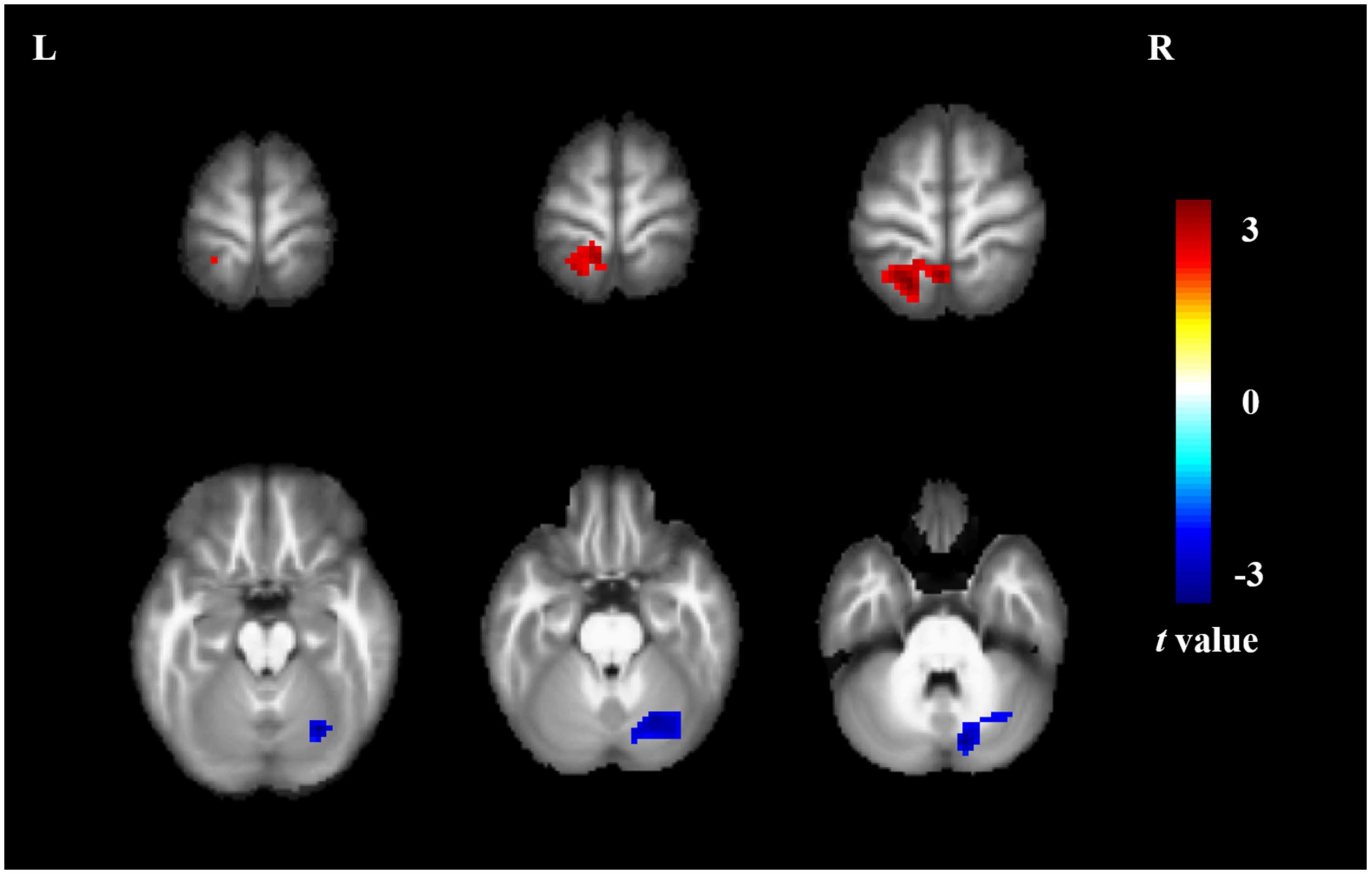
ReHo comparison between the elderly with short sleep duration and non-short sleep duration. The figure shows the two-sample t-test parameter diagram corrected by Alphasim. The warm color in color bar indicates that the ReHo value of the elderly with short sleep duration is higher than that of the non-short sleep duration group, whereas the cool color is the opposite. The ReHo values of left superior parietal gyrus increased in the elderly with short sleep duration. The ReHo values of right crebellum decreased in the elderly with short sleep duration.

Compared to non-short sleep duration group, short sleep duration group showed significantly increased ReHo value in the left superior parietal gyrus, and decreased ReHo value in the right crebellum (*p* < 0.05, AlphaSim correction, cluster size >55 voxels) (Figure 2 and Table 3).

**Table 3:** Brain region information of ReHo value.

Brain regions	Cluster size	Cluster centroid MNI coordinates			*t*-value
		x	y	z	
Left superior parietal gyrus	124	−18	−54	72	3.614
Right cerebellum	156	12	−78	−30	−3.739

MNI: Montreal Neurological Institute.

### 3.4. DC analysis

**Figure 3:**
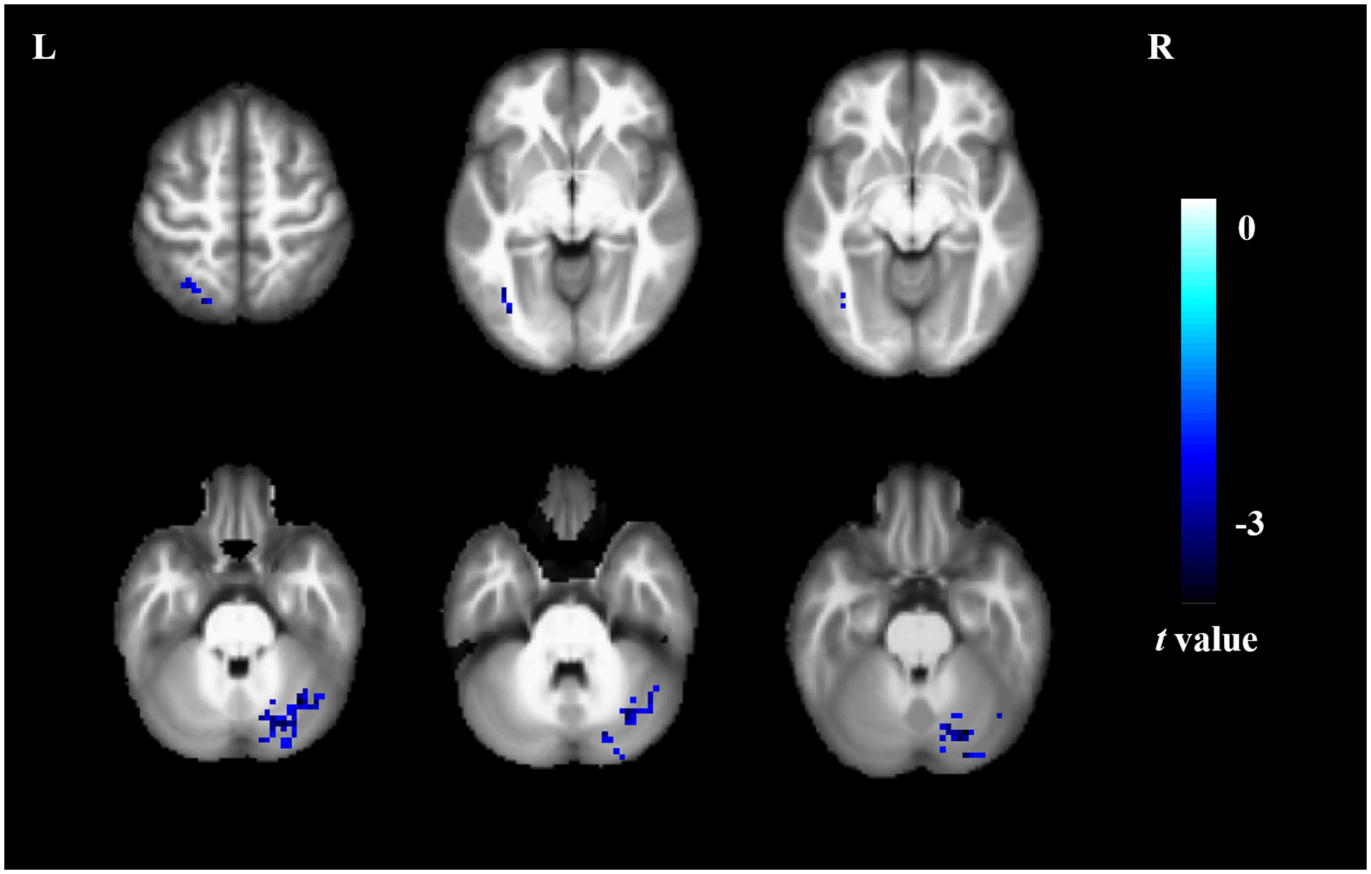
DC comparison between the elderly with short sleep duration and non-short sleep duration. The figure shows the two-sample t-test parameter diagram corrected by Alphasim. The cool color in color bar indicates that the DC value of the elderly with short sleep duration is lower than that of the non-short sleep duration group. The DC values of left inferior occipital gyrus, left superior parietal gyrus and right cerebellum decreased in the elderly with short sleep duration.

Compared to non-short sleep duration group, short sleep duration group showed significantly decreased DC value in the left inferior occipital gyrus, left superior parietal gyrus and right cerebellum (p < 0.05, AlphaSim correction, cluster size >14 voxels) (Figure 3 and Table 4).

**Table 4:** Brain region information of DC value.

Brain regions	Cluster size	Cluster centroid MNI coordinates			*t*-value
		x	y	z	
Right cerebelum	107	24	−75	−27	−3.890
Left inferior occipital gyrus	21	−39	−63	−6	−3.162
Left superior parietal gyrus	24	−21	−72	57	−3.148

MNI: Montreal Neurological Institute.

### 3.5. Correlation among clinical data, fMRI and cognitive function

To investigate the correlation between the age/education/BMI/sleep duration and the ALFF/ReHo/DC value, a general linear model was fitted with the ALFF/ReHo/DC value as the dependent variable and the age/education/BMI/sleep duration as independent variables. Table 5 shows the results of significant correlation. Another general linear model was fitted to assess the independent association between the ALFF/ReHo/DC value and SDMT/STT_B score, the ALFF value of right insula significantly associated with SDMT score (*β* = −0.363, *p* = 0.033).

**Table 5:** Correlation between clinical data and fMRI.

	ALFF value			ReHo value		DC value	
	β1	β2	β3	β4	β5	β6	β7
age	0.131	0.130	−0.008	0.023	0.011	0.051	0.074
BMI	−0.046	−0.039	−0.055	0.023	−0.057	0.010	−0.152
education	0.305^*^	0.237	−0.016	−0.071	0.087	−0.191	0.002
sleep duration	−0.438^**^	−0.424^*^	−0.430^*^	0.519^**^	−0.532^**^	0.534^**^	0.544^**^

^*^p < 0.05; ^**^ *p* < 0.001; 1, Left middle frontal gyrus; 2, Right middle frontal gyrus; 3, Right insula; 4, Right cerebellum; 5, Left superior parietal gyrus; 6, Right cerebellum; 7, Left inferior occipital gyrus.

## 4. Discussion

Herein, we characterized spatial patterns of intrinsic brain activity and functional connectivity in the elderly with short sleep duration, and explored the relationship between spatial patterns of intrinsic brain activity and information processing speed, Interestingly, we found that short sleep duration and processing speed are significantly associated with remodeling spatial patterns of intrinsic brain activity in the elderly. Our findings point to therapeutic targets for improving processing speed in in the elderly with short sleep duration.

ALFF with a typical frequency range (0.01 Hz ~ 0.08 Hz) is considered to describe the local spontaneous neuronal activity, which is thought to be physiologically meaningful (Ma et al., 2021). The elderly with short sleep duration showed increased spontaneous neuronal activity in the bilateral middle frontal gyrus and right insula in the study. Insomniacs are often accompanied by depression. Studies have also found the abnormally spontaneous neural activity in the middle frontal gyrus in patients with depression (Fu et al., 2021). In addition to abnormal brain function, the decrease of gray matter volume in the frontal lobe was also found in insomniacs with impaired attention executive function (Joo et al., 2013). Increased ALFF value of insular is also often found in patients with insomnia (Wu et al., 2021). These studies are consistent with our findings.

ReHo based on Kendall’s coefficient concordance was developed to measure the temporal synchronization of the time series of a given voxel and compare it with its nearest neighbors in a voxel-wise data-driven way (Yang et al., 2020). We found that the elderly with short sleep duration showed increased ReHo value in the left superior parietal gyrus, and decreased ReHo value in the right crebellum. It is reported that the emotional perception index was negatively correlated with ReHo value in the left superior parietal gyrus, and the left superior parietal gyrus could compensate for facial emotion recognition in patients with bipolar disorder (Hwang et al., 2021). After taking antidepressants, the ReHo value is decreased in the left superior parietal gyrus in patients with major depressive disorder (Wang et al., 2014). Abnormal ReHo value in the right cerebellum contribute to anger dysregulation (Gao et al., 2021). A prospective study of chronic primary insomnia suggested a lower ReHo value in the right cerebellum, which is consistent with our findings (Dai et al., 2014).

In addition, we found that the elderly with short sleep duration showed significantly decreased DC value in the left inferior occipital gyrus, left superior parietal gyrus and right cerebellum. Lee et al. reported that there are significant characteristic differences in inferior occipital gyrus function between patients with psychogenic insomnia and normal individuals (Dai et al., 2014). It was also found that the change of DC in the inferior occipital gyrus was significantly related to the change of sleep quality in the other diseases (Fan et al., 2022). Previous studies have found that the decrease of parietal cortical nerve activity in sleep deprived groups (Javaheripour et al., 2019). Insomnia is closely related to glucose metabolism in parietal lobe (Kay et al., 2016). Chou et al. found that changes of grey matter volumes and decrease of structural covariance integrity was observed in cerebellum in insomniac patients (Chou et al., 2021). In conclusion, the superior parietal gyrus is closely associated with emotion, and our findings may explain a mechanism for brain remodeling in the emergence of depressive states in the elderly with short sleep duration.

Interestingly, we found the ALFF value of right insula significantly associated with SDMT score, which point outs short sleep duration reduces processing speed via remodeling spatial patterns of intrinsic brain activity and functional connectivity in the elderly. The insula plays an important role in perception, emotion, and self-awareness. Specifically, it has four main functions: 1. Processing and integrating interoceptive information: the insula is a crucial region in the brain that encodes many internal bodily sensations such as heart rate, blood pressure, and digestion. It processes and integrates interoceptive information, helping us better understand the relationship between the body and mind. 2. Generating diverse experiences and behaviors: different insula neurons can respond differently to the same internal signals, which can contribute to generating diverse experiences and behaviors in different contexts. 3. Participating in emotional regulation: the insula is also involved in emotional regulation, influencing emotional experiences and expressions. 4. Participating in the generation of self-awareness: the insula is related to the generation of self-awareness, particularly in relation to bodily self-awareness. These functions are critical to our understanding of the relationship between the body and mind (Namboodiri and Stuber, 2020). Age can affect the processing speed and accuracy of decision-making. As people grow older, their processing speed may decrease, which could potentially impact their decision-making abilities. Time perception is another factor that influences decision-making. People’s perception and understanding of time can affect their decision-making process (Löckenhoff, 2011). The right insula plays an important role in information processing speed. Specifically, it is involved in negative emotional responses, which may affect an individual’s processing speed and accuracy of emotional information. Additionally, the right anterior insula is also involved in the attention network, which can regulate an individual’s attention allocation and shifting, and therefore may affect the processing speed and accuracy of attention. These findings suggest that the right anterior insula plays a critical regulatory role in large-scale neural networks, and has a significant impact on the processing speed and accuracy of both emotional and attentional information (Touroutoglou et al., 2012). Our research contributes to a deeper understanding of the neural mechanisms underlying the relationship between sleep and attention in the elderly population. Our finding also provides stimulation targets for the elderly with short sleep duration, as well as valuable insights into new approaches to improve sleep functions via neuromodulation technology.

## 5. Limitations

This study also had several limitations. First, the sample size is small, we will expand the sample size to verify the results of this study. Second, we lack objective indicators in evaluating the quality of sleep, we will add sleep monitoring for further in-depth analysis of the relationship between sleep quality and brain function. Third, multimodal fMRI analysis can be performed in subsequent studies, and longitudinal studies also need to be investigated in subsequent phases.

## 6. Conclusion

Short sleep duration and processing speed are significantly associated with remodeling spatial patterns of intrinsic brain activity in the elderly.

## Data availability statement

The raw data supporting the conclusions of this article will be made available by the authors, without undue reservation.

## Ethics statement

The studies involving human participants were reviewed and approved by the local ethics committee at the fourth people’s hospital of Chengdu. The patients/participants provided their written informed consent to participate in this study.

## Author contributions

G-JY and LP: conceptualization and writing–original draft. YZ: methodology. YW and J-LL: validation. X-NZ and XZ: formal analysis. LP: writing, review and editing. All authors contributed to the article and approved the submitted version.

## Funding

This work was supported by Chengdu Municipal Health Commission (no. 2022277).

## Conflict of interest

The authors declare that the research was conducted in the absence of any commercial or financial relationships that could be construed as a potential conflict of interest.

## Publisher’s note

All claims expressed in this article are solely those of the authors and do not necessarily represent those of their affiliated organizations, or those of the publisher, the editors and the reviewers. Any product that may be evaluated in this article, or claim that may be made by its manufacturer, is not guaranteed or endorsed by the publisher.

## Footnotes

^1^ http://www.fil.ion.ucl.ac.uk/spm
